# Affinity Electrophoresis of Proteins for Determination of Ligand Affinity and Exploration of Binding Sites

**DOI:** 10.3390/ijms26073409

**Published:** 2025-04-05

**Authors:** Patrick Masson, Tatiana Pashirova

**Affiliations:** 1 Institute of Fundamental Medicine and Biology, Kazan Federal University, 18 Kremlyovskaya St., 420008 Kazan, Russia; tatyana_pashirova@mail.ru; 2 Arbuzov Institute of Organic and Physical Chemistry, FRC Kazan Scientific Center of RAS, Arbuzov Str. 8, 420088 Kazan, Russia

**Keywords:** affinity electrophoresis, affinity capillary electrophoresis, dissociation constant, polyacrylamide gel, protein–ligand interactions

## Abstract

Affinity gel electrophoresis was introduced about 50 years ago. Proteins interact with a ligand immobilized in the support. Specific interactions cause a decrease in electrophoretic mobility. The presence of a free ligand, competing with an immobilized ligand, restores electrophoretic mobility. In affinity capillary electrophoresis, the ligand is mobile, and its interaction with a specific protein changes the mobility of the protein–ligand complex. This review mostly focuses on gel affinity electrophoresis. The theoretical basis of this technique, ligand immobilization strategies, and principles for determination of ligand affinity are addressed. Factors affecting specificity and strength of interactions are discussed, in particular, the structure of the affinity matrix, pH, temperature, hydrostatic pressure, solvent, co-solvents, electric field, and other physico-chemical conditions. Capillary affinity electrophoresis principles and uses are also briefly introduced. Affinity gel electrophoresis can be used for qualitative and quantitative purposes. This includes detection of specific proteins in complex media, investigation of specific interactions, protein heterogeneity, molecular and genetic polymorphism, estimation of dissociation constants of protein–ligand complexes, and conformational stability of binding sites. Future prospects, in particular for screening of engineered mutants and potential new drugs, coupling to other analytical methods, and ultra-microtechnological developments, are addressed in light of trends and renewal of this old technique.

## 1. Introduction

The fantastic development of electrophoretic and isoelectric focusing (IEF) techniques in the fourth quarter of the last century allowed simple and reliable determination of protein size, overall shape and geometry, conformational stability, and functional parameters of proteins [1]. In particular, gel electrophoresis helped understand the biochemical polymorphism of proteins, their different electric charges, conformations, and quaternary structures, thus becoming a simple and reliable criterion of protein purity, macromolecular organization, and functionality. Affinity electrophoresis (AE), the term coined by Bøg-Hansen [2], offered the possibility of exploring specific surface interactions and quantifying interactions between binding sites and ligands. In particular, affino–immuno-electrophoresis techniques were initially developed by the group of Bøg-Hansen [3,4] for investigating the microheterogeneity of glycans covalently attached to glycoproteins and to quantify their interactions with lectins (glycan-/sugar-binding glycoproteins). A related approach allows for the investigation of antibody–antigen reactions [5] and protein–protein interactions [6]. Analysis of specific interactions between protein binding sites and small ligands was independently developed by Nakamura and Takeo in Japan [7,8,9] and Ticha and Hořejší in Czechoslovakia [10,11,12,13]. Later, affinity capillary electrophoresis (ACE), also called capillary zone affinity electrophoresis (CZAE), approaches utilized minute amounts of proteins, which increased the resolution and rapidity of analyses, leading to high-throughput screening of ligand libraries [14,15,16,17].

While AE was mostly developed between the 1970s and the end of the 1990s, ACE (CZAE) started to develop after 1990 and rapidly expanded. While more than 4000 articles on ACE have been published, those about gel AE have progressively decreased. The most recent review comparing gel AE and ACE was published 15 years ago [18], and the last review on new AE techniques was published 10 years ago [19]. However, in the past decade there was a renewal of interest in gel AE [20], in particular with the development of 1D and 2D immobilized metal AE [21] and high-throughput immuno-affinity electrophoresis on microfluidic cards for determination of binding affinity of recombinant antibody libraries [22]. Moreover, classical gel AE does not need the implementation of complex apparatuses, and it is much more sensitive than ACE, specifically in enzyme activity detection, leading to the formation of colored precipitates in the gel matrix. Thus, taking into account new technological developments of AE, it was of interest in the present review to mostly address principles of polyacrylamide gel affinity electrophoresis (PAG-AE), implementation, and achievements of AE for investigating protein–ligand interactions in biochemistry, pharmacology, immunology, and biotechnology. The basic principles of ACE are not different from those of gel AE. Although ACE is now regarded as one of the main analytical methods for exploring drug–protein interactions and determining binding parameters [23]. We only briefly reviewed ACE and highlighted its significant applications for studying protein–ligand interactions.

## 2. Fundamental Principles of AE

**Scheme 1:**
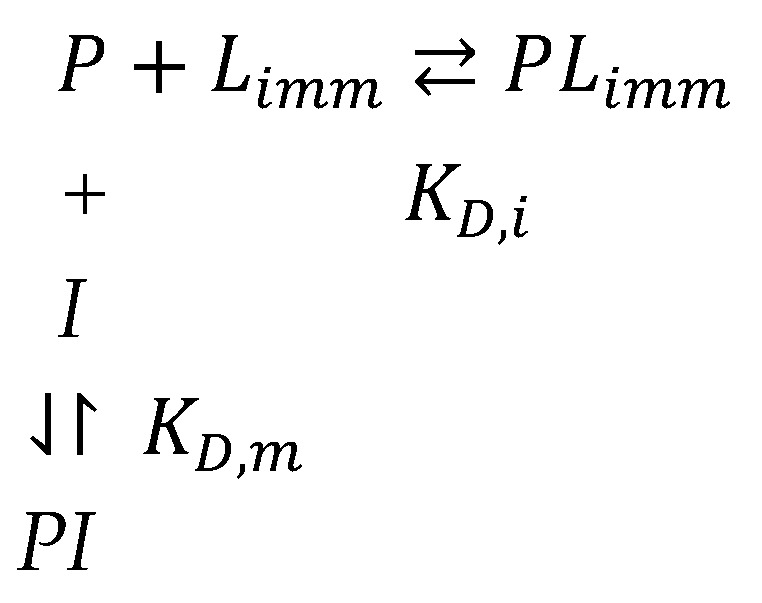
Minimum model for the interaction of a protein (*P*) with an immobilized ligand (*L_imm_*) in the presence of a non-immobilized competing ligand (*I*). The affinity of a protein P for *L_imm_* and mobile competing ligand (*I*) is expressed either as a dissociation constant (*K_D,i_* and *K_D,m_*) of complexes *PL*_imm_ and *PI* or as an association constant (*K_A,i =_ K_D,i_* ^−1^ and *K_A,m =_ K_D,m_^−^*^1^) (Equations (1) and (2)), where *i* stands for “immobilized ligand” and *m* for “mobile ligand”.

Several electrophoretic techniques can be referred to as AE. These techniques apply the principles of biospecific interactions to zone electrophoresis in a gel or a capillary tube. In gel AE, proteins (*P*) migrate into a medium that contains immobilized ligands (*L_imm_*) in the presence or absence of free competing ligands (*I*) (Scheme 1). In ACE, ligands are not immobilized but free in the buffer phase. Here, we focus on gel AE; theoretical principles of ACE will be developed in the ACE section. Specific and reversible interaction of *P* with *L_imm_* leads to a decrease in the electrophoretic mobility of *P*.

Then, under given electrophoretic conditions, the mobility of *P* depends on its affinity of *P* for *L_imm_* and its effective concentration in the medium. The presence in the medium of a non-immobilized, free competitor of *L_imm_*, i.e., *I*, impairs the interaction of *P* with *L_imm_* and progressively restores the initial mobility of *P* as [*I*] is increasing.

(1) $$K_{D}=\frac{\left[P\right]\left[L\right]}{\left[PL\right]},$$

(2) $$K_{A}=\frac{\left[PL\right]}{\left[P\right]\left[L\right]},$$

The first works describing specific interactions between proteins and immobilized ligands (macro-ligands) in electrophoretic matrices were published at the end of the 60 s. Entlicher et al. [24] observed that the mobility of lectins on starch gel is increased in the presence of sugars incorporated in the matrix: sugars interfered with specific interactions of lectins with the starch matrix. The first application of polyacrylamide gel (PAG) AE to quantify interactions between isoenzymes of phosphorylase and immobilized glycogen was published more than 50 years ago [25,26].

## 3. Methodology of Gel AE

We will describe the methodological principles of AE in slab gels and in gel rods. As regards CZAE and AE in capillary tubes, the methodology is different and will not be elaborated on here. Unlike gel AE, in CZAE, ligands are not immobilized ligands but are free molecules injected into the capillary with the protein sample.

### 3.1. Immobilization of Ligands in Matrix

Gel AE was developed using different types of matrices for immobilization of ligands: starch, agarose, PAG, and mixed gels of acrylamide–agarose and acrylamide–starch. A major issue in AE is complete immobilization of the ligand in the matrix. Ligand immobilization techniques depend on both the matrix and the type of ligand. Two main approaches can be used: (Ia) the ligand is derived first, i.e., a chemical modification of the ligand with a functional group enables it to react in situ with the polymeric matrix; (Ib) a chemically modified ligand reacts with the monomeric units of the future matrix before its polymerization, where a 3D ternary block copolymer is formed; (II) a ligand is polymerized first into a “macro-ligand”, which is mixed with monomeric units of PAG, which are then left to polymerize. In the case of agarose, a macro-ligand is added to a melted matrix. The ternary cross-linked functionally derived PAG was first used to study the interaction of lectins with sugars [27,28].

At first allyl-glycosides were synthesized, and affinity gels were prepared by radical copolymerization of these ligands with acrylamide and bis-methylene acrylamide monomers. However, the co-polymerization yield was low due to the poor reactivity of allyl derivatives, which also inhibit acrylamide polymerization [29]. These experiments involved tedious work, where, after the formation of ternary copolymers in gel rods, it was necessary to uncast gels and extensively wash them with electrophoresis buffer before reintroducing them into electrophoresis tubes. Although the synthesis of 3D functionalized PAGs is the most appealing for making homogenous gels where ligands are covalently immobilized to the matrix, it is poorly reproducible. Hence, it cannot be utilized for quantitative studies, and it is difficult to implement. However, in affinity trap PAGE, a qualitative affinity technique related to Western blot, the ligand can be conveniently coupled to a linker (e.g., *N*-succinimidyl 3-(2-pyridyldithio)propionate (SPDP)) prior to the copolymerization with acrylamide and bis-acrylamide to make the affinity slab gel matrix used for orthogonal electro-transfer of proteins [30].

In quantitative AE, a macro-ligand is usually trapped in the matrix. This is carried out by mixing linear macro-ligands with acrylamide monomers before triggering the polymerization of acrylamide, which results in homogenous affinity gels. Alternatively, heterogeneous gels can be formed by embedding within the PAG network of polymeric beads where the ligand was previously grafted.

**Figure 1:**
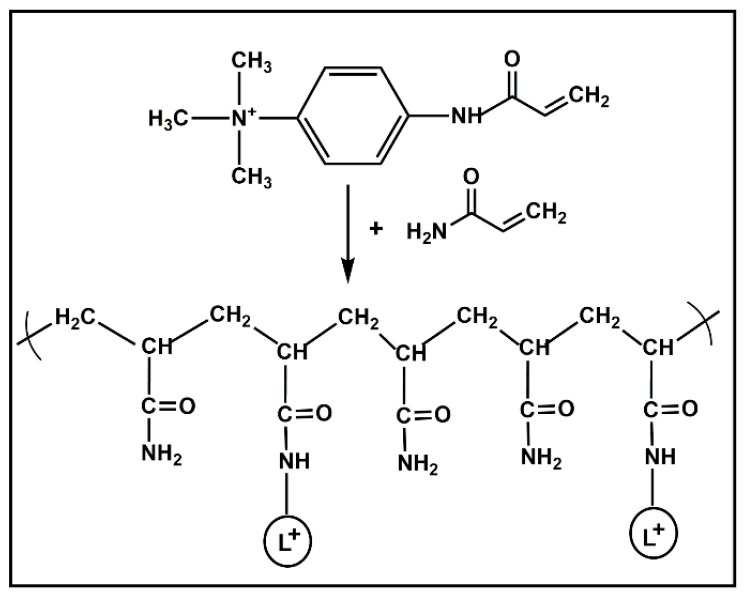
A reversible inhibitor of cholinesterases, acryloyl derivative of p-amino-phenyl-trimethylammonium as a functional acrylamide derivative for making an altered linear copolymer of acrylamide–ligand or macro-ligand for PAG-AE of ChEs [41]. A macro-ligand supporting m-amino-phenyl-trimethyl ammonium acryloyl derivative was also synthesized for the same purpose [42].

Different types of macro-ligands have been described in the literature: (a) natural polymers acting as natural substrates or ligands of enzymes such as starch for amylases [31,32], glycogen for phosphorylases [25,26,33], blood group glycoproteins as ligands of carbohydrate-binding proteins such as lectins [34], denatured proteins for proteases [35], nucleic acid for nucleases [35,36]; (b) High molecular weight dextrans chemically modified to carry specific ligands, e.g., blue dextran for protein interacting with Cibacron Blue F-3GA [37], p-aminobenzamidine bound to Dextran T-500 for trypsin [38], p-aminophenyl glycosides bound to Dextran T-500 for lectins [39], hydrophobic chains for phosphorylases [7]. Before conjugation to dextran, ligands must be activated by periodic acid oxidation [38] or reaction with CNBr [7]. (c) Linear polyacrylamide macro-ligands for AE can be synthesized by copolymerization of acrylamide with allyloyl [11,28] or acryloyl derivatives of ligands (Figure 1) [40,41,42,43]. The latter are more reactive than allyloyl derivatives, leading to longer monodisperse copolymers that are easier to immobilize in PAG.

**Figure 2:**
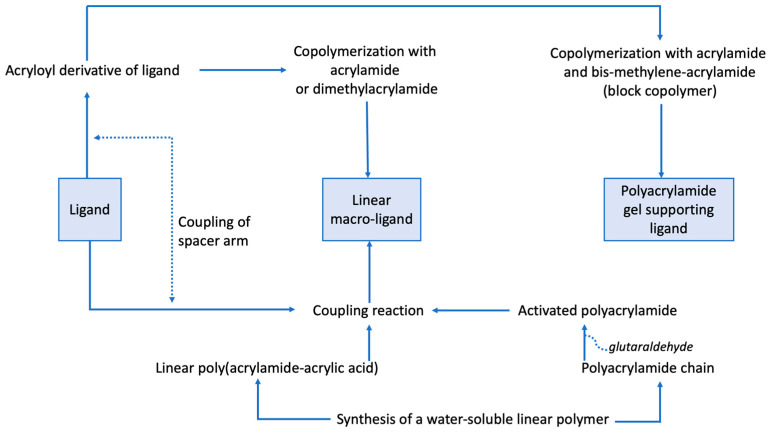
Methods for derivation and immobilization of ligands in PAG.

Alternatively, linear copolymers bearing free carboxyl groups can be prepared first and then coupled to functionalized ligands. For instance, copolymers of alternating acrylamide and acrylic acid can be coupled to amino derivatives of ligands via a water-soluble carbodiimide [44]. After coupling, residual carboxylic groups can be neutralized. Reactive linear copolymers of N-(2-hydroxypropyl) methacrylamide and esters of N-methacryloyl ω-amino acids have also been used [37]. Long polyethylene glycol derivatives can serve as carriers for ligands. For instance, intercalating phenylphenazinium dyes were linked to PEG chains for AE study of DNA fragments [45]. (d) The last approach is immobilizing ligands on insoluble matrices like agarose, dextran, or polyacrylamide beads, but, in this case, matrices must first be activated. Agarose and dextrans can be activated by periodic oxidation or by reacting with cyanogen bromide or epoxide derivation. Polyacrylamide is activated by glutaraldehyde or hydrazine [11]. The different strategies for immobilization of ligands in PAG were summarized in [46]. Figure 2 shows these different methods for immobilization of ligands.

### 3.2. Electrophoretic Conditions

All techniques of mono- and bi-dimensional gel electrophoresis and IEF can be implemented in AE. For quantitative measurements, it is important to control the different parameters of the electrophoretic system: gel composition and porosity, effective concentration of free and immobilized ligands, buffer pH, conductivity and ionic strength, temperature, and electric field.

**Figure 3:**
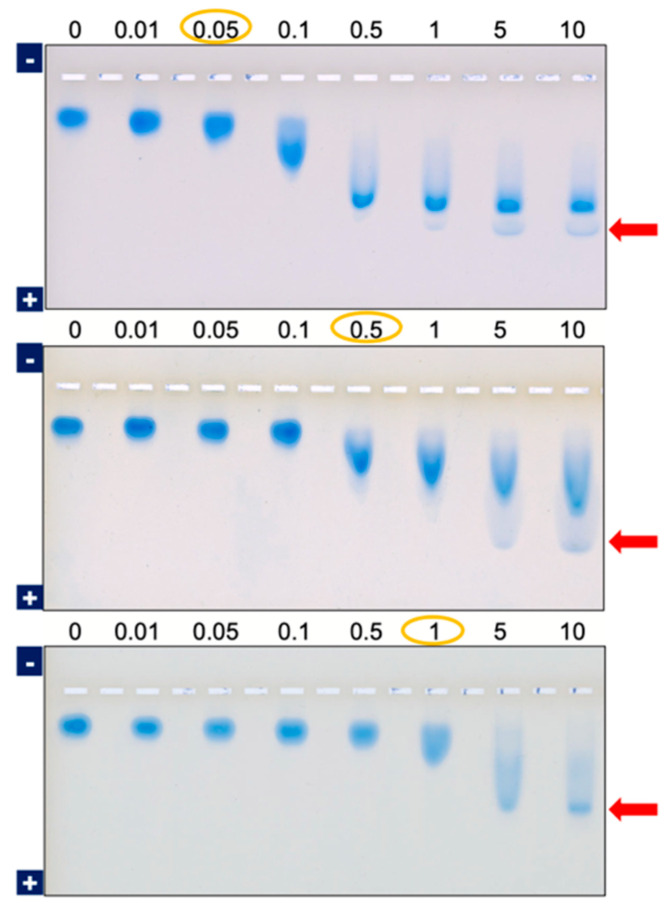
Bovine serum albumin (BSA, 3 μg, pI = 4.7) mobility on 1% agarose gels in the presence of increasing detergent concentration (%): the upper panel, sodium dodecyl sulfate, SDS; the middle panel, Sarkosyl; and the lower panel, sodium lauroyl-glutamate, SLG. Buffer system: 0.1 M His/0.1 M MES at pH 6.1. BSA was stained with Coomassie Brilliant Blue. Red arrows indicate fast moving bands that may correspond to new BSA complexes (from [53]). Reproduced with permission of Elsevier.

The most widely used technique is AE in discontinuous PAG systems based on disk-electrophoresis in gel rods [47], which can be of standard diameter (5 mm) or less (3 mm or 1 mm). The concentration of acrylamide in PAGs is defined by two parameters: the total concentration of acrylamide monomers (bis-acrylamide crosslinker + acrylamide) calculated per 100 mL (%T), and %C, the reticulation concentration of gel (bis-acrylamide/%T). While the macro-ligand is embedded in separating gel, the free ligand is present in all components of the electrophoretic system, including the migration buffers. In classical disk-electrophoresis, a stacking gel is located above the separating gel. Due to the discontinuous buffer system in disk-electrophoresis, proteins from samples concentrate during the passage through the stacking gel and enter the separating gel as a thin disk. Therefore, the protein concentration compared to the concentration of immobilized ligand may not be negligible, complicating the AE process and making the quantitative interpretation of results more challenging. It is generally better to omit stacking gels for AE. AE in slab gels is an alternative to disk-PAGE for multiple protein samples, which can migrate under the same pH and ligand concentration [48]. The advantage of the slab gel technique is a rapid comparison of the binding affinity of multiple mutants (natural or engineered) of a single protein. Other gel electrophoresis techniques can be implemented in the presence of immobilized ligands, which we will briefly mention here: isotachophoresis [49]; IEF in a tube or on a slab gel, where the formation of an adduct between protein and immobilized ligand causes a pI shift of the protein, which can be reversed in the presence of a free competitive ligand [11]. The 2D variant introduced by Righetti’s team [33,50] is an extension of the electrophoretic titration curve approach on a PAG plate in the presence of a macro-ligand [51]. This technique enables assessment of how dissociation/association constants for protein–ligand interactions depend on pH. Affinophoresis, or mobile AE, is a qualitative technique introduced by Shimura and Kasai [52]. It is a qualitative technique that utilizes a mobile macro-ligand called “affinophore” embedded in a high-porosity gel such as agarose gel. The affinophore is a dextran of small molecular weight affinity bearing a large number of charged groups in addition to multiple specific ligand units. The protein specifically interacts with specific ligand units. The proteins carrying the same electric charge as an affinophore pass through such gels with increased mobility. A variant of this technique is electrophoresis in 1% agarose gel pre-loaded with various charged detergents. Depending on the binding affinity of proteins for detergents, the concentration of detergents, and pH of the running buffer, the mobility of the moving proteins changes, e.g., it is increased if pH > pI. Beyond a specific detergent concentration, the denaturing effect of the bound detergent on protein can be observed as a smear. The curve of mobility versus increased detergent concentration adopts a sigmoidal form [53] (Figure 3).

## 4. Applications of AE

The different variants of AE provide simple and potent tools for qualitative and quantitative study of protein–ligand interactions. These techniques are of interest for the following: the detection and identification of specific proteins in complex media; the detection of molecular and genetic polymorphism (isoenzymes and allelozymes); the control of homogeneity of purified proteins; the investigation of molecular and functional heterogeneity of proteins (usually following their isolation and purification); the studying of the effects of chemical modifications of proteins; and the determination of the apparent dissociation constant of proteins. If AE is conducted under varying temperatures or pressures, it can be used to assess the apparent thermodynamic parameters such as free energy of binding and volume change upon binding.

### 4.1. Quantitative Applications

By using AE, we can determine apparent dissociation constants of a protein for an immobilized ligand *(K_D,i_*) or free (mobile) ligands (*K_D,m_*), even with minute amounts of protein. The affinity of proteins for a ligand can also be expressed by binding constants (*K_A_*) instead of dissociation constants (*K_A_* = *K_D_*^−1^). *K_D_* is determined by measuring the migration distances (*m*) (or relative migration distances (*Rm*) of the studied protein versus the distance traveled by the tracking dye (or, alternatively, a protein not interacting with ligands) on gels containing increasing amounts of an immobilized ligand in the presence or absence of mobile ligands. The principle is similar to quantitative affinity chromatography for determination of protein binding constants [23]. However, AE has a higher resolution power and precision and much higher sensitivity than affinity chromatography.

#### 4.1.1. Theoretical Background

Takeo and Nakamura [26] were the first to state the principles of quantitative AE. However, most theoretical developments came from the Czech group [49,54,55,56].

The determination of *K_D_* by AE is based on three postulates: (i) the association and dissociation rates of a protein–ligand complex are bigger than the migration velocity (electrophoretic mobility) of the free protein in gel; (ii) the mobility of the ligand in the protein–ligand complex is zero; and (iii) the concentration of the immobilized ligand is very high compared to the protein concentration in the migrating zone [*L*] >> [*P*], so that the total concentration of ligand [*L*] is approximately equal to the concentration of the free (unbound) ligand [*l*]:

(3) $$\left[l\right]=\left[L\right]-\left[PL\right]\approx \left[L\right],$$

In the case of monovalent interactions (one molecule of protein binds one ligand molecule):

(4) P+L⇄PL,

(5) $$K_{D,i}=\frac{\left[P\right]\left[L\right]}{PL},$$

Then, the fraction of free protein ∅ (concentration of free protein compared to total concentration of protein) is equal to the ratio of electrophoretic mobility in the presence of ligand (*Rm_i_*) over the mobility in the absence of ligand (*Rm_o_*):

(6) $$\emptyset =\frac{\left[P\right]}{\left[P\right]+\left[PL\right]}\equiv \frac{{\left[P\right]}_{free}}{{\left[P\right]}_{total}}=\frac{{Rm}_{i}}{{Rm}_{0}},$$

then,

(7) $${Rm}_{i}^{-1}={Rm}_{0}^{-1}\left(1+\frac{\left[L\right]}{K_{D,i}}\right),$$

**Figure 4:**
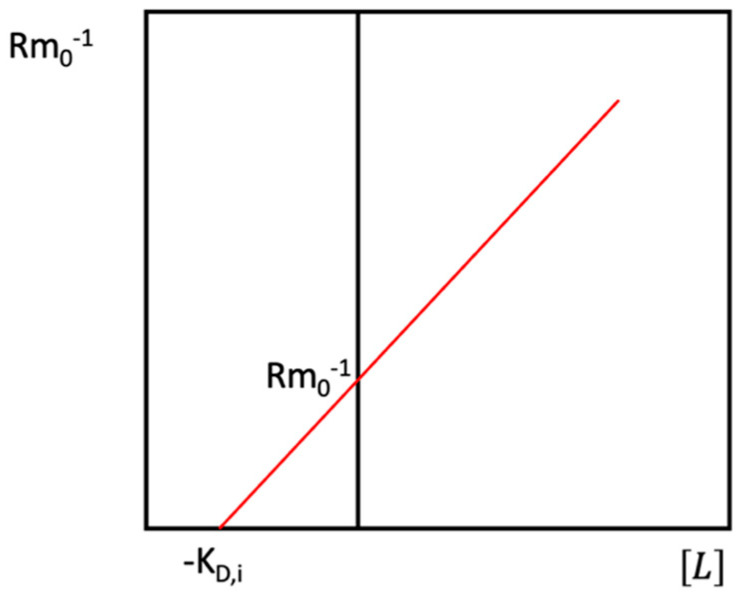
Takeo–Nakamura plot for graphical determination of *K_D,i_* from relative mobility change as a function of immobilized ligand concentration [*L*] (Equation (7)).

Equation (7), initially derived by Takeo and Nakamura [26], allows us to graphically determine *K_D,i_* (Figure 4).

When a free (mobile, *m*) ligand, *I*, competing with the immobilized ligand, *L_imm_*, is present in the medium, we have to consider both equilibria described in Scheme 1. Then, the 4th postulate of AE is to state that the mobility of complexes *PI* is identical to the mobility of the free protein, and the 5th postulate is that the concentration of free mobile ligand [*i*] is approximately equal to the total concentration of mobile ligand [*I*], with [*I*] >> [*P*]:

(8) $$\left[i\right]=\left[I\right]-\left[PI\right]\approx \left[I\right],$$

Then,

(9) $$\emptyset =\frac{{Rm}_{i}}{{Rm}_{0}}=\frac{\left[P\right]+\left[PI\right]}{\left[P\right]+\left[PI\right]+\left[PL\right]},$$

and,

(10) $$r=\frac{{Rm}_{i}}{{Rm}_{0}-{Rm}_{i}}=\frac{K_{D,i}}{\left[L\right]}\left(1+\frac{\left[I\right]}{K_{D,m}}\right),$$

**Figure 5:**
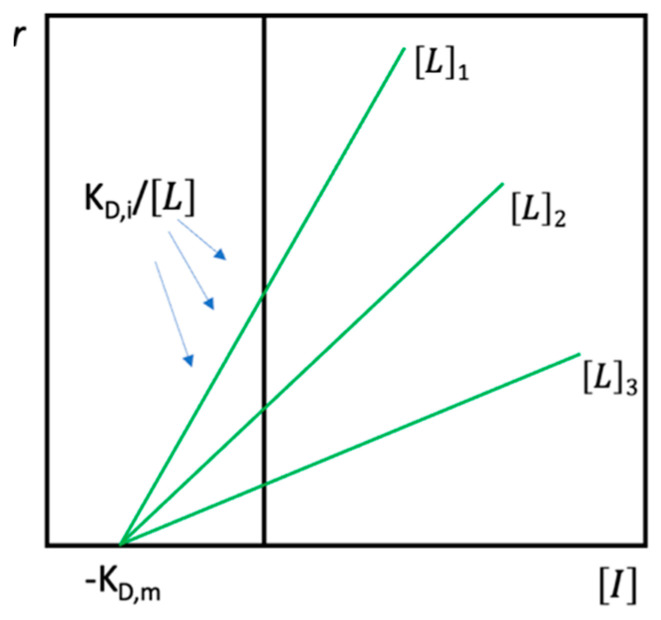
Graphical determination of *K_D,i_* and *K_D,m_* from relative mobility change as a function of concentration of mobile ligand [*I*] (Equation (10)) and three different concentrations of immobilized ligand [*L*].

Equation (10), initially proposed by Hořejší [54], allows simultaneous graphical determination of *K_D,i_* and *K_D,m_*, using one concentration of immobilized ligand and several concentrations of mobile ligand (Figure 5).

#### 4.1.2. Factors Affecting Values of K_D_ in AE

Although *K_D_* values determined by AE are, in general, in agreement with values calculated from kinetic and equilibrium experiments, it is important to confirm these values since apparent *K_D_* may be strongly affected by experimental conditions. The following describe the factors affecting quantitative affinity parameters.

AE performed under extreme physical conditions [1] allows us to assess the effect of physical and chemical variables on protein affinity and/or to evaluate the effect of dissociation/aggregation of proteins on that affinity. AE may help reveal conditions causing the loss of protein functional properties in early unfolding steps of protein unfolding or those involved in the improved conformational stability of functional structures in the presence of additives or co-solvents. For these purposes, AE can be performed under altered physical and physicochemical conditions: high hydrostatic pressure (*P*), different temperatures (*T*) including subzero-temperatures, different pH and varying electric field intensity, different solvents (e.g., buffered H_2_O versus buffered D_2_O), in the presence of organic co-solvents or non-charged denaturing agents (e.g., urea), or stabilizers (e.g., polyols) added to electrophoresis buffers.

- Temperature

The dependence of dissociation (*K_D_*) or association (*K_A_*) constants (*K*^−1^*_D_* = *K_A_*) on temperature can be determined by performing AE at different temperatures, e.g., from −5 °C to 60 °C, in a thermally controlled electrophoresis apparatus. The effect of temperature (*T*) on an equilibrium constant is described by the Van’t Hoff equation (Equations (11)–(13)):

(11) $${\left(\frac{{\partial LnK}_{D}^{-1}}{\partial (\frac{1}{T})}\right)}_{P}=-\Delta H/R,$$

(12) $${LnK}_{D}^{-1}=-\frac{\Delta H}{RT}+\frac{\Delta S}{R},$$

(13) $${LnK}_{D}^{-1}=-\frac{\Delta G}{RT},$$

**Figure 6:**
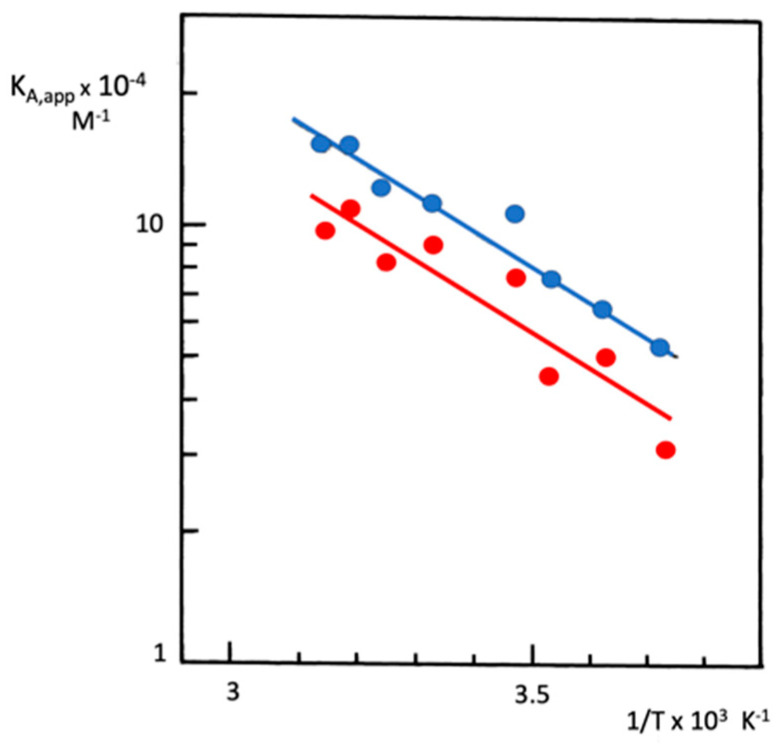
Van’t Hoff plot (Equation (12)) for the temperature dependence of native and chemically modified human butyrylcholinesterase (BChE) with immobilized procainamide. (●), native tetramer; (●) soman-aged tetramer in which the active serine (S198) is phosphonylated [44]. The OP soman is methyl-pinacolyl phosphonofluoridate; after enzyme phosphonylation on S198, the “aging” reaction leads to dealkylation of the pinacolyl group on the OP adduct, and the resulting aged enzyme is methyl phosphonyl enzyme. Figure adapted from [44]; reproduced with permission of Elsevier.

In Equations (11)–(13), Δ*H*^0^, Δ*S*^0^, and Δ*G*^0^ are enthalpy change, entropy change and free energy change upon ligand binding, respectively. R is the gas constant (1.986 cal/K·M). Then, apparent thermodynamic parameters of ligand binding can be determined from Van’t Hoff diagrams [44,57]. Thermodynamic parameters determined by AE are comparable to those determined by other methods, such as equilibrium dialysis and fluorescence quenching [58]. AE also detects subtle differences in conformation/hydration between wild-type and mutant enzymes or between native and chemically modified proteins. These differences affect the binding affinity of these proteins for the respective specific ligand. For instance, Van Hoff plots of data obtained from PAG-AE of native vs. soman-aged BChE for a reversible ligand such as immobilized procainamide (Figure 6) showed a small difference in the binding affinity and free energy of binding difference between the two enzyme forms (ΔΔ*G°_app_* = 0.22 kcal·M^−1^). In addition, there was no difference between the two enzyme forms regarding the enthalpy change upon the procainamide binding (Δ*H°_app_* = 3.5 kcal·M^−1^). However, a small but significant increase in entropy (Δ*S°_app_* = 0.8 e.u.) not only suggested a hydration change but also a decrease in plasticity of the soman-aged enzyme binding site in comparison to the native enzyme, which was later confirmed by conformational stability, X-ray crystallography, and neutron scattering studies [59,60,61].

- Hydrostatic pressure

To study the conformational equilibrium of a protein [1] and to determine the volume changes (Δ*V*) accompanying the formation of the protein–ligand adduct, we can utilize the plot depicting pressure (*P*) dependence of binding constants, which can be studied by performing AE under high hydrostatic pressure (up to several kbar) in massive high-pressure vessels at controlled temperature (for descriptions on apparatuses, see [62]). In Equation (14), *T* is the absolute temperature and *R* the gas constant (= 82 mL atm K^−1^ M^−1^; 1 atm = 1.013 bar = 105 Pa). The magnitude and sign of Δ*V* provide information on the nature of binding interactions, solvation state, and microenvironment of binding sites [63,64].

(14) $${\left(\frac{{\partial LnK}_{D}^{-1}}{\partial P}\right)}_{T}=-\Delta V/RT,$$

**Figure 7:**
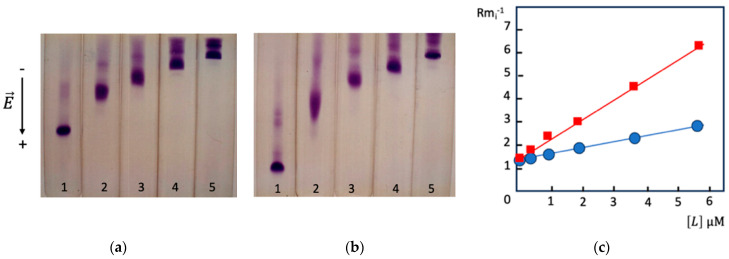
Affinity electrophoresis of bovine serum albumin (BSA) on a copolymer of linear polyacrylamide and immobilized octadecyl ligand. The band of BSA monomer is the fastest migration band, while the BSA oligomers are slower. AE at the atmospheric pressure (**a**) and under high hydrostatic pressure of P = 1.8 kbar (**b**), both at 35 °C. (**c**) Takeo plots: red line, AE at atmospheric pressure; blue line, AE under P = 1.8 kbar. The immobilized ligand concentrations (μM) in gels were as follows: 0 (lane 1); 0.90 (lane 2); 1.81 (lane 3); 3.62 (lane 4); and 5.43 (lane 5). Adapted from an original figure in [43]; reproduced with permission of Wiley.

Hence, AE represents a versatile tool for exploring affinities of protein binding sites, their topology, and the stability of proteins. Furthermore, AE carries the potential to be employed to study recently discovered extremo-proteins (from hyperthermophiles and piezophiles) and protein mutants of unknown 3D structures. For instance, AE under rising hydrostatic pressure (from the atmospheric pressure of 1 bar up to 1.8 kbar) at different temperatures (5–35 °C) on PAG containing an immobilized octadecyl ligand was used to probe the interaction of fatty acid-free bovine serum albumin (BSA) with a long-chain aliphatic ligand (N-acryloyl-6-amidocaproic acid N-octadecylamide copolymerized with N,N,-dimethylacrylamide) [43] (Figure 7).

**Figure 8:**
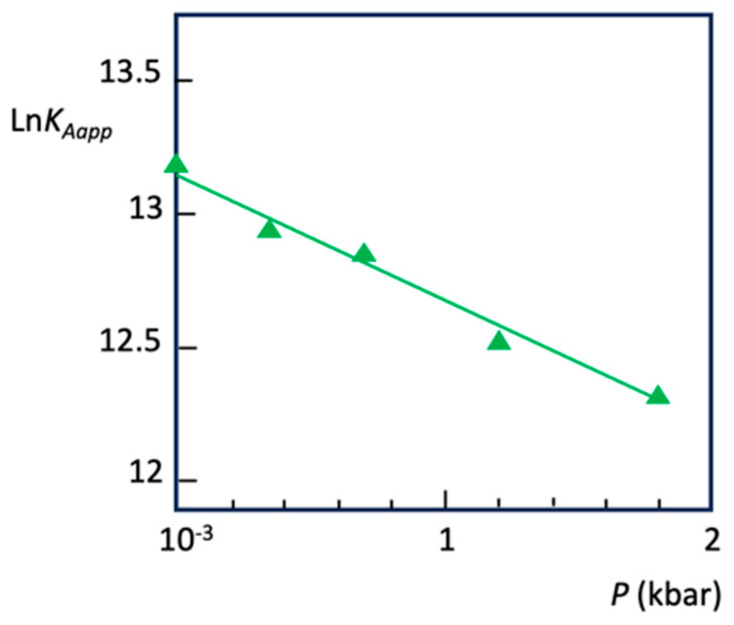
The apparent association constant (*K_A_*) of BSA interaction with the immobilized octadecyl ligand is pressure-dependent at 35 °C. The slope = −Δ*V/RT*. Adapted from [43]; reproduced with permission of Wiley.

The increased pressure results in lower binding affinity of BSA for the octadecyl ligand (Figure 7c). The volume change at constant temperature corresponding to the binding of octadecyl chains to BSA was calculated from Equation (14). Figure 8 shows how apparent *K_A_* alters with pressure at 35 °C, enabling the calculation of Δ*V*, which is +11.6 mL·M^−1^.

**Figure 9:**
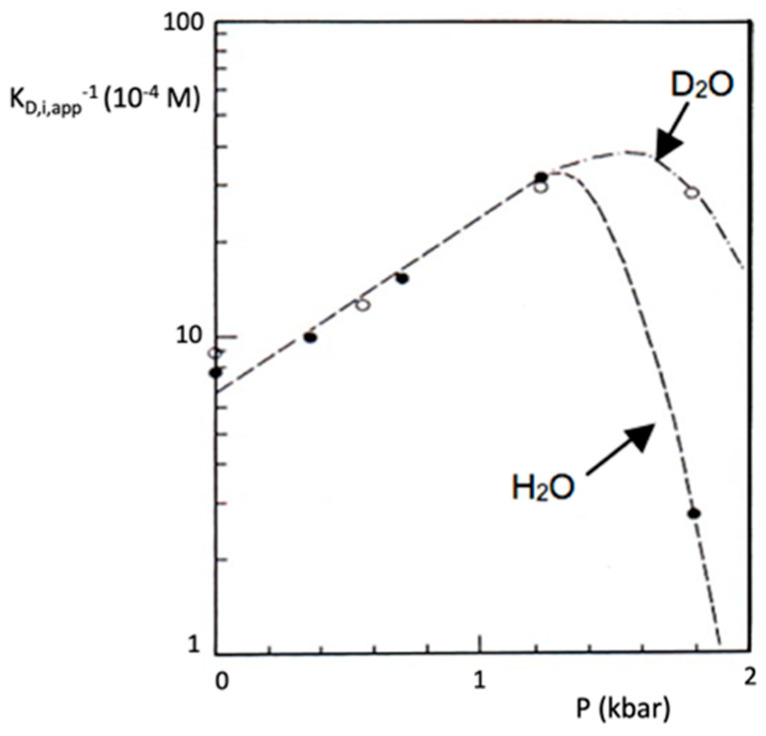
Increase in binding (b) affinity of human butyrylcholinesterase (BChE) for immobilized p-amino-phenyl trimethylammonium as a function of the rising pressure at 35 °C (Δ*V_b_* = −Δ*V_KD_* = −33 mL·M^−1^) up to 1.3 kbar. Note a drop in affinity in a narrow pressure interval [65]. Reproduced with permission from Elsevier.

As seen in Figure 8, pressure decreases the binding affinity of BSA for the hydrophobic ligand, i.e., Δ*V* > 0. The linearity of the plot shows that no compressibility change occurs in the pressure range. This means that the rising pressure does not induce conformational change in BSA long-alkyl chain binding sites, at least not in the pressure range up to 1.8 kbar. The volume change is positive due to the fact that hydrophobic interactions are accompanied by the release of water molecules that were associated with hydrophobic surfaces (hydrophobic solvation). Thus, hydrophobic interactions are unfavored by the rising of pressure. In contrast, aromatic ring stacking or π-cation interactions between the protein and its ligands are favored by the rising pressure and are accompanied by negative volume changes (Δ*V* < 0) [63,64]. The next example (Figure 9) illustrates this case.

As seen in Figure 9, the pressure strengthens (Δ*V* < 0) the binding affinity of BChE for the immobilized phenyl trimethyl ammonium ligand, thus suggesting the presence of an aromatic ring involved in the binding site. Indeed, later it was demonstrated by X-ray diffraction (crystallography) that the residue tryptophan, W82, resides in the binding site [66]). In a buffer prepared with heavy water (D_2_O) instead of water, there is no solvent isotope effect on binding up to 1.3 kbar. However, beyond 1.3 kbar, the affinity rapidly drops in both solvents. This drop in affinity corresponds to a pre-unfolding transition, i.e., transient formation of a molten globule state [1]. Because of the pressure stabilization of deuterium bonds compared to hydrogen bonds, pressure-induced loss in affinity is retarded by about 200 bars in heavy water.

- Solvents, co-solvents, stabilizers, and denaturing agents.

A recent review has already covered the presence of additives in running buffer, acting on the conformation and structural stability of proteins [1]. These additives, stabilizers (e.g., polyols) or denaturants (e.g., urea), must be non-charged molecules. AE can be performed in 1D AE-gel rods in the presence of different concentrations of additives or in 2D PAGE, such as in transverse urea gradient gel electrophoresis, containing a single concentration of an immobilized ligand [1]. The importance of solvents in AE was clearly shown/demonstrated in Figure 9 [67], where the solvent–isotope effect of deuterium stabilizes the functional protein structure against pressure-induced unfolding. Similarly, the presence of 2M co-solvent sorbitol in electrophoresis buffers stabilizes the quaternary structure of the BChE tetramer against pressure-induced dissociation up to 2 kbar [62].

- pH.

pH of the running buffer firmly controls proteins’ electrophoretic mobility [1] but may affect the strength of interactions. The effect of pH on *K_D_* is assessed by performing AE at different pH [38,68], giving rise to an electrophoretic affinity titration curve, which appears to be the most straightforward technique [33,50]. When a protein–ligand interaction strongly depends on pH, AE has to be carried out in a continuous instead of a discontinuous buffer–gel system, such as in disk-electrophoresis, where pH varies. Continuous buffer–gel systems are a must-use with ligands bearing ionizable groups involved in interaction with protein binding sites. An example is a ligand with a functional group whose pK_a_ = 9.3 and whose deprotonated form cannot bind to the studied protein. Then, the classical discontinuous buffer system of Ornstein, performed at pH = 8.3 [47], cannot be used because during electrophoretic migration, the pH of the running gel will increase to 9.5 and lead to deprotonation of half of the ligand functional groups. This, in turn, would lead to a drop in the effective ligand concentration in this buffer system, thus reducing the apparent strength of interaction. The most useful continuous and discontinuous buffer systems for PAGE have been reviewed [69,70], and all of them can be used in gel-AE.

- Ionic strength.

Ionic strength (*I*) (Equation (15)) of electrophoretic buffers is defined as follows:

(15) $$I=\frac{1}{2}\sum_{i}c_{i}z_{i}^{2},$$

where *i* is the number of ionic species in the buffer, *c*_i_ is their concentration, and *z_i_* is their charge. It must be relatively low to minimize ohmic heating [71,72]. In practice, in electrophoresis, ionic strength can vary only over narrow intervals [1], and according to the Debye–Hückel–Onsager law, its optimal values must range from 10 to 100 mM. The study of the interaction of neutral immobilized ligands, such as carbohydrates, with lectins [73] showed that ionic strength between 11 and 90 mM has no significant effect on the strength of interaction. On the contrary, when proteins interact with charged ligands, the buffer’s ionic strength effect on binding affinity has to be considered [41,74].

- Electric field.

The effect of an electric field ($\vec{E}$) on a binding equilibrium can be described by Equation (16):

(16) $${\left(\frac{{\partial LnK}_{D}^{-1}}{\partial E}\right)}_{T,P}=-\Delta M/RT,$$

where Δ*M* is the molar difference in macroscopic dielectric moment between P and PL. Thus, applying an external electric field may alter the strength of interactions between proteins and ligands and alter the protein conformational stability [75].

Although there were few reports on electro-desorption in AE [75], it is known that the electric field may affect protein conformation and alter its binding properties [76]. This effect is likely negligible in gel AE, but it could be significant in CAE, where the electric field can reach over 500 V/cm. Despite this, it was assumed that, for current uses of CAE, the electric field does not affect the binding affinity [77] of proteins. The electric field may have different effects on different experimental systems. When macro-ligands are charged, the electric field may modify the dipolar moments of interacting groups, the protein conformation, and the gel structure [78]. In addition, the electric field may act through the Joule effect and the consecutive effect of heat on binding constants.

- Gel porosity: interactions between protein and PAG matrix, containing immobilized ligand.

The importance of gel porosity on protein–macro-ligand interactions has to be taken into account.

Theoretical and experimental data indicated that both the 3D structure of the gel and the mobility of the protein through the polymer network contribute to favoring interactions in denser gels [13,79,80]. Let us consider homogenous affinity gels, containing a macro-ligand in which immobilized ligand molecules are randomly distributed within the gel matrix and equally accessible to the migrating protein. We may apply the model where the protein migration in the gel is a succession of random collisions with ligand molecules that are chemically equivalent. Then, protein–macro-ligand interactions can be calculated in terms of event probability [79].

Experimentally, important changes in the apparent affinity of proteins for immobilized ligands as a function of [*T*] were observed. For example, the apparent *K_D_* of BChE molecular forms for procainamide decreased with the increase in %*T*, i.e., when gel porosity decreases [81]. In PAG-AE, where [*L*] and [*T*] vary, the differential of relative mobility (*dR*_m_) is expressed as follows:

(17) $$dRm={\left(\frac{\partial R_{m}}{\partial T}\right)}_{L}dT+{\left(\frac{\partial R_{m}}{\partial L}\right)}_{T}dL,$$

Then, the *K_D,app_* as a function of %*T* can be derived and graphically determined via the following equation:

(18) $$K_{D,app}=\left[L\right]/\left[\left(exp(-Ln10\Delta K_{R}T\right)-1\right],$$

where *K_R_* is the retardation coefficient, which depends on the size of the protein [82]. Apparent affinity, *K_Dapp_*, for immobilized ligands linearly increases with %*T* until an optimum %*T_opt_*. In other words, the probability of interaction increases with %*T*, i.e., with the decrease in gel porosity until the point where optimal interactions occur.

In conclusion, the gel structure imposes physical constraints whose consequences are as follows: (a) a protein may witness a restricted access to a fraction of immobilized ligand molecules, which may cause a drop in the effective ligand concentration, and (b) altered probabilities of different events in the temporal sequence, collision without interception, collision with interception (binding), altered lifetime of protein–ligand complexes, and interval between two collisions. The parallel evolution of the probability of one of these events with rising %T compensates for the opposite effects of increasing %*T* on the availability of ligand to the migrating protein.

- Protein binding affinity characteristics

The size of the protein, its monomeric or oligomeric structure, the number of binding sites (*n*), and its concentration in the migrating zone determine the mobility and, consequently, the values of apparent *K_D,i_* and *K_D,m_*.

**Figure 10:**
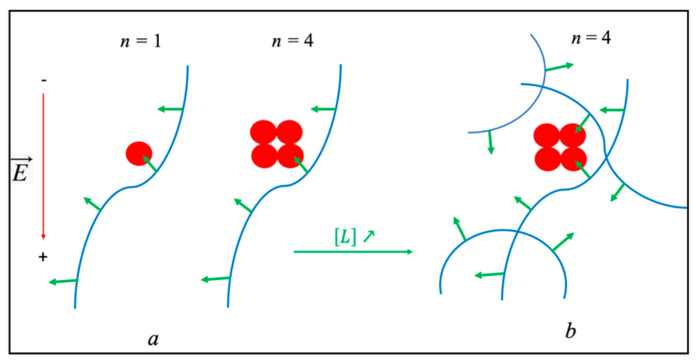
AE of a monomeric (*n* = 1) and a tetrameric (*n* = 4) protein. It is assumed that immobilized ligand molecules are statistically distributed within the gel matrix: (**a**) monovalent interactions at low [*L*] and (**b**) bivalent interactions at high [*L*].

Let us consider a monomeric protein with a single binding site (*n* = 1) and an oligomeric protein with *n* ≥ 2. Regarding the latter, the following two situations are possible: (a) if the size and conformation/shape of the migrating protein along with the 3D structure of the immobilized ligand in the gel permit the binding of this ligand to only one binding site (out of *n*), then the situation may be considered equivalent to a monovalent interaction, i.e., a single ligand binds to a single binding site on the protein monomer. In this case, dissociation constants are *K_D,i app_* = *K_D,i_*/*n* (Figure 10a); (b) if several sites on the protein can simultaneously attach to the immobilized ligand, then the probability of simultaneous interactions of several sites depends on a probability, *p* (0 < *p* < 1). The *p* is a Poisson function of the protein’s spatial geometry and the availability of immobilized ligand in the affinity gel. In the case of bivalent interactions (Figure 10b), *p* depends on the distance, *d*, between two binding sites on the protein and the immobilized ligand expressed as the number of effective functional groups per unit volume [56]:

(19) $$p=1-e^{\left(-4\pi d^{3}\left[L\right]/3\right)},$$

**Figure 11:**
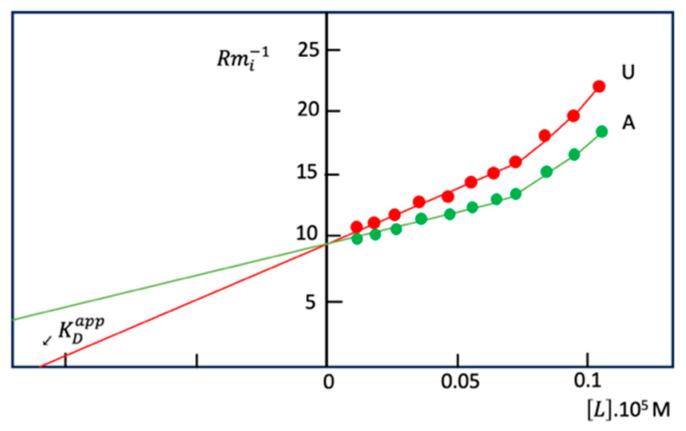
The curvature of Takeo–Nakamura plots at high concentrations of an immobilized ligand due to multiple interactions in oligomeric proteins. The change in the electrophoretic mobility of tetrameric BChE originating from two different homozygous phenotypes, U (●) and A (●), is plotted versus [*Limm*], thus providing the calculation of *K_Dapp_* = 0.1·10^−4^ M and 0.19·10^−4^ M for U and A, respectively. Adapted from an original figure in [42]; reproduced with permission of Elsevier.

Moreover, gel porosity, depending on %T, also affects the probability of monovalent and multivalent interactions. Thus, in the case of multivalent interactions (Figure 10b), Takeo–Nakamura plots presented in Figure 11 show a curvature [41,42,56]. Graphical determination of *K_D,i_* is possible from the linear portion of plots.

**Figure 12:**
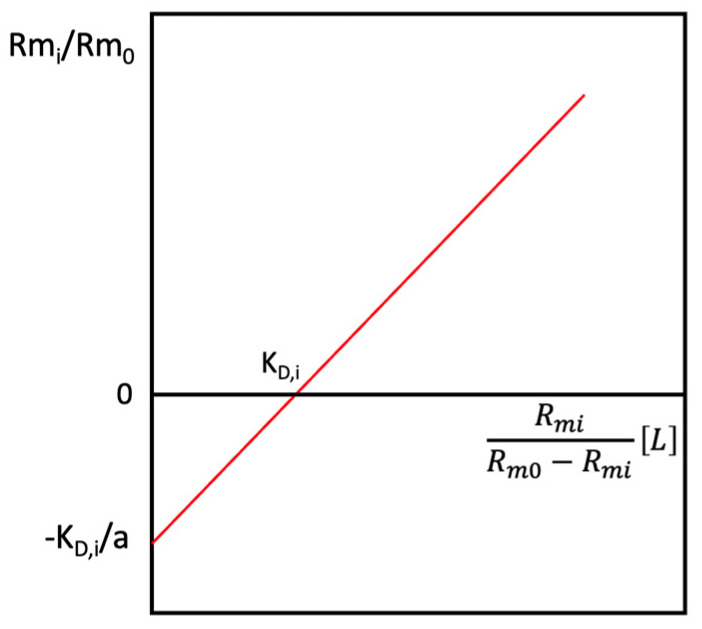
Determination of dissociation constant for immobilized ligand when the protein concentration (a) in the migration zone is not negligible compared to [*L*].

Regarding multivalent proteins bearing *n* equivalent binding sites, where several sites (*n*’ ≤ *n*) interact simultaneously with the immobilized ligand, *n*, *n*’, and *K_D_* cannot be calculated from curved AE plots [13]. However, the quantitative analysis is even more complicated when the binding sites are non-equivalent. Let us consider the protein concentration (*a*) in the migrating zone. If *a* is not negligible compared to [*L*], the classical Takeo plots are not linear. For such cases, Hořejší and colleagues derived equations [48,56] that allow graphical determination of apparent *K_D,i_* from linear plots (Equations (19) and (20) and Figure 12):

(20) $$\frac{{Rm}_{i}}{{Rm}_{0}-{Rm}_{i}}=\frac{1}{{\left[L\right]}_{eff}}\left(\frac{{Rm}_{i}}{{Rm}_{0}}a+K_{D,i}\right),$$

Or

(21) $$\frac{{Rm}_{i}}{{Rm}_{0}}=\frac{1}{a}\left(\frac{{Rm}_{i}}{{Rm}_{0}-{Rm}_{i}}{\left[L\right]}_{eff}-K_{D,i}\right),$$

In Equations (19) and (20), [*L*]_eff_ is the effective concentration of immobilized ligand. Although the value of [*L*]_eff_ is a priori different from analytical [*L*], it can be estimated by monitoring the change in electrophoretic mobility as a function of *a* in a continuous AE buffer system at constant [*L*] [56]. *K_D_* of a complex formed by a protein and a mobile ligand is dependent neither on the immobilized ligand nor on [*L*]_eff_. Thus, determination of *K_D,m_* only needs knowing [*I*] present in gel. It can be determined even in the case of very weak affinity where *K_D,m_* ≈ 10^−1^ M [54]. It must be noted that when mobile ligands are charged, determination of *K_D,m_* is erroneous [37,41]. Thus, in such cases, gel AE is not operative, and *K_D,m_* must be determined using alternatives biophysical methods for micro-samples, e.g., ACE or classical ligand-binding methods used in enzymology for investigating reversible ligands/inhibitors.

The assumption that mobility of a P-L_imm_ complex is zero is not valid when macro-ligands are incompletely immobilized (e.g., due to charged macro-ligands or small macro-ligands) so that they can travel alongside the bound protein through the gel. Such complications were considered by others [48]. For both cases, graphical determinations of *K_D,i_* were proposed [13].

#### 4.1.3. Time-Dependent Interactions

In principle, forming a reversible complex is very fast; it occurs in a range of a few micro-seconds. However, slow formation and dissociation of complexes are also possible in AE, such as slow-binding inhibitors in solution [83,84]. In this case, the symmetry and width of moving zones provide information on such slow kinetic processes. It was demonstrated that if the half-time of the protein–ligand complex is shorter than 100–1000 times the duration of the electrophoresis run, kinetic effects are negligible. On the other hand, if this half-time is 10–100 shorter, then the moving zone widens asymmetrically [48]. Then, densitometric analysis of asymmetric electrophoretic zone profiles after staining, as for analysis of electrophoretic profiles of P and P-L complexes in AC (see Section 5), allows assessment of kinetic constants of ligand binding and dissociation [85]. Practically, the rate constants for the formation and dissociation of a P-L complex can be determined from the half-width of the protein band, but only in the case of proteins with a single binding site (*n* = 1) [79].

Although enzyme kinetic analysis, surface plasmon resonance, and isothermal titration calorimetry are more suitable for determination of binding constants and other kinetic and thermodynamic variables, the main advantage of AE is its usage of minute amounts of biological material.

**Scheme 2:**
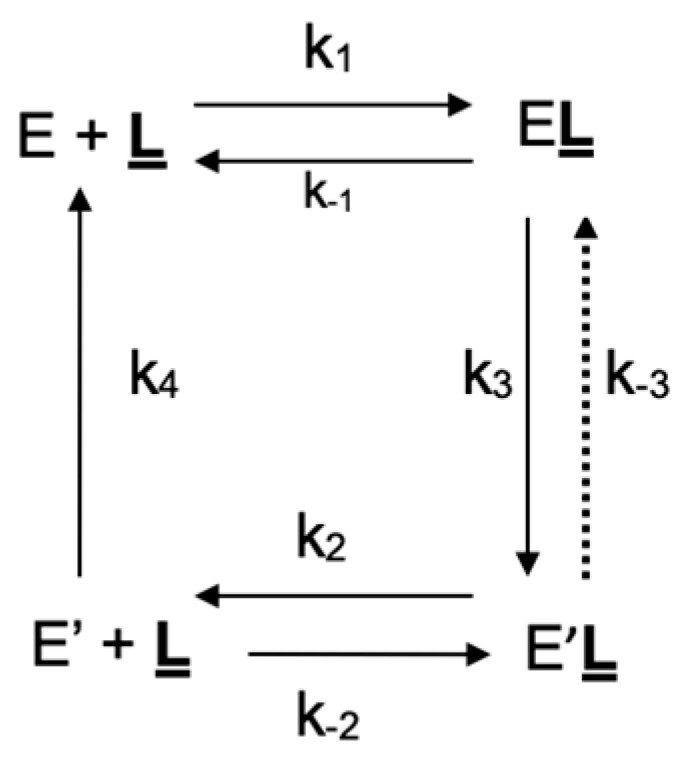
Model for immobilized ligand (*L*)-induced long-lived isomeric enzyme form E’.

During AE, a time-dependent reversible process can be observed. For example, in AE of cholinesterases (ChEs) on immobilized reversible inhibitors (e.g., phenyl trimethyl ammonium and procainamide) [41,42,44], it was found that above a critical immobilized ligand concentration (L), depending on the gel concentration [81], a slow enzyme migrating form (zone) (E’) appeared and intensified at the expense of the initial enzyme form (E). The slow migrating form was interpreted as the long-lived ligand-induced BChE isomer [86] (Scheme 2).

In Scheme 2, *K_D_* = k_-1_/k_1_, *K’_D_* = k_-2_/k_2_, and *K’_D_* < *K_D_*. The reactions characterized by rates k_-3_ and k_4_ are slow compared to the rapid association and dissociation steps *k_2_* + *k_-2_* of L. At low L, the enzyme form is E, and the enzyme progressively is converted into the E’ form as the concentration of immobilized ligand increases. Binding of E to immobilized ligand L induces a discrete isomerization of the enzyme (*k_3_*), causing an increase in affinity of E’ for L, i.e., *K’_d_* < *K_d_*. Because the slow form predominates at high L, it means that the reverse reactions (*k_-3_* and *k_4_*) E’→E are slow compared to the rapid association/dissociation process (*k_2_* + *k_-2_*) of the migrating enzyme interacting with L.

**Scheme 3:**
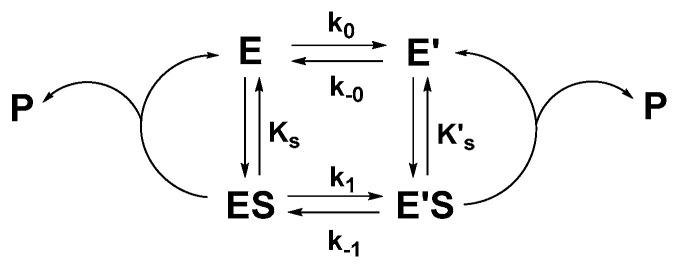
General Frieden model for hysteretic enzymes. *k*_0_ and *k*_−0_, and *k*_1_ and *k*_−1_ are the kinetic constants of the slow equilibria E⇆E’ and ES⇆E’S, respectively.

However, in light of the hysteretic behavior of ChEs in catalytic and inhibition processes [86,87], an alternative explanation can be proposed. Assuming that the enzyme exists as two forms, E and E’, co-existing in a slow equilibrium, the general Frieden mechanistic model for hysteretic catalytic behavior [88] (Scheme 3) can account for the phenomenon observed in AE of human BChE with immobilized ligands.

This pattern of protein interaction with reversible ligands has also been explained by the slow-binding inhibitor (SBI) mechanism of type C [89]. Accordingly, the fact that the lowest affinity form (E) prevails at low L implies that *k_-_*_0_ > *k*_0_. Then, because *K’_d_* < *K_d_*, the equilibrium is progressively shifted to E’. Because the reaction with rate constant *k_4_* in Scheme 2 (equivalent to the rate constant *k*_-0_ in Scheme 3) is slow compared to the enzyme electrophoretic mobility, the enzyme remains in the E’ state between two consecutive hits with immobilized ligand (L = ↑) as pictured in Figure 10.

#### 4.1.4. Interactions Between Gel Matrix and Macro-Ligand

The immobilization of the ligand and its effective concentration (accessibility) in the affinity matrix depend on the characteristics of the matrix (e.g., %*T* of PAG), the nature of the polymer carrying the ligand, the spacers, and the chemical structure of the ligand.

The conformation of a macro-ligand adopted in a solution depends on the macro-ligand backbone and the type of its substituent(s). In solution, linear PA chains form random coils with a few helix segments. The presence of substituents may favor polymer structuration [90]. However, the conformation of such linear macromolecules trapped in PAG is different. The interpenetration of both polymeric chains causes changes in the spatial organization of the AE matrix compared to simple PAG. Indeed, the complete immobilization of certain macro-ligands of low molecular mass within PAG cannot be simply explained by their physical trapping in the gel network [39,45,91]. It seems that multiple non-covalent bonds, and in some cases, covalent bonds are formed between PA and macro-ligands. Moreover, certain macro-ligands may induce opacity of PAG [54] or may inhibit the polymerization of acrylamide [37]. Gelfi and Righetti showed that turbidity of PAGs reflects heterogeneous distribution of PA chains, causing altered gel porosity [92]. Thus, in AE gels, immobilized ligand molecules may be non-homogeneously distributed, making analytical and effective concentrations of immobilized ligand different. As a consequence, it is difficult to estimate the effective concentration of immobilized ligand.

The influence of the macro-ligand structure on the accessibility of ligands to proteins, and hence, on apparent values of *K_D_*, was first reported in the case of lectin–glycoside interactions [39].

On the other hand, *K_D_* strongly depends on the spacer length [38,40,91]. As a rule, the affinity of a protein for the ligand increases with the spacer length until an optimal number of methylene groups.

### 4.2. Qualitative Applications

#### 4.2.1. Detection of Specific Proteins in Complex Media

In theory this technique of “fishing” in complex media allows the identification of specific proteins from their retardation in AE gels. The specificity of interaction can be controlled by operating in AE gels that contain free competitor ligands. However, the presence of multiple competing proteins and/or the low specificity of ligands may impair this technique. Thus, specific enzymes can be detected either in gels where a specific macromolecular substrate is trapped or after simple PAGE followed by Western electro-blotting. For the first approach, the following two methods can be used: (a) the enzyme migrates in gels at a pH far below the optimum enzyme reaction pH, so that no reaction occurs during migration. Then, after migration, gels are subjected to a buffer with a pH that is optimal for the enzyme activity in the presence of a chromogenic substrate; then, the enzyme reaction occurs and zones of activity progressively develop; (b) alternatively, run AE under optimal pH in the presence of a colored substrate trapped in the gel. The migration distance of the enzyme is obtained by measuring the length of the negatively stained part of the gel, i.e., the distance where the colored substrate was degraded by the enzyme during its migration. This method was successfully applied to detect amylases in starch-enriched gels, proteases in gels containing specific protein substrates for proteases, and nucleases in gels containing nucleic acids or synthetic polynucleotides [35]. The activity of migrating enzymes can be compared by changing the nature of immobilized substrates or by incorporating competitive inhibitors in gels. For the approach involving Western blotting, the proteins present in a complex medium are first separated by standard slab PAGE, then electro-transferred/captured on an affinity gel, and finally, transferred to a PVDF membrane for subsequent analysis, e.g., by mass spectrometry of proteolytic fragments [30].

#### 4.2.2. Detection of Protein Heterogeneity and Genetic Polymorphism

Detection of allelozymes and isoenzymes of the same size and charge but different affinity for specific ligands can be carried out by AE. The technique was initially implemented by Hořejší et al. [54] for the detection of isolectins on gels containing copolymers of acrylamide and allyl sugar derivatives. Isoenzymes of lacticodehydrogenase (LDH) [37] or alcohol dehydrogenase [93] can be detected in a similar way on gels containing Blue Dextran. Allozymes of human BChE, originating from point mutations that affected the binding affinity of this enzyme for positively charged ligands, can also be detected this way. Figure 11 illustrates the different affinity of purified human BChE homozygous phenotypes U (E_1_^u^E_1_^u^) and A (E_1_^a^E_1_^a^) for immobilized m-phenyl-trimethyl ammonium due to a single point mutation (A70D) hitting the enzyme peripheral binding site. Heterozygous enzyme of phenotype UA shows an intermediate affinity for the respective ligand [42]. However, although AE works well with purified allelozymes and may be used to discover the respective genotypes (determined by PCR), in complex media such as human plasma or cellular extracts, the presence of multiple competing proteins impairs the sensitivity of AE, thus restricting its applicability.

**Figure 13:**
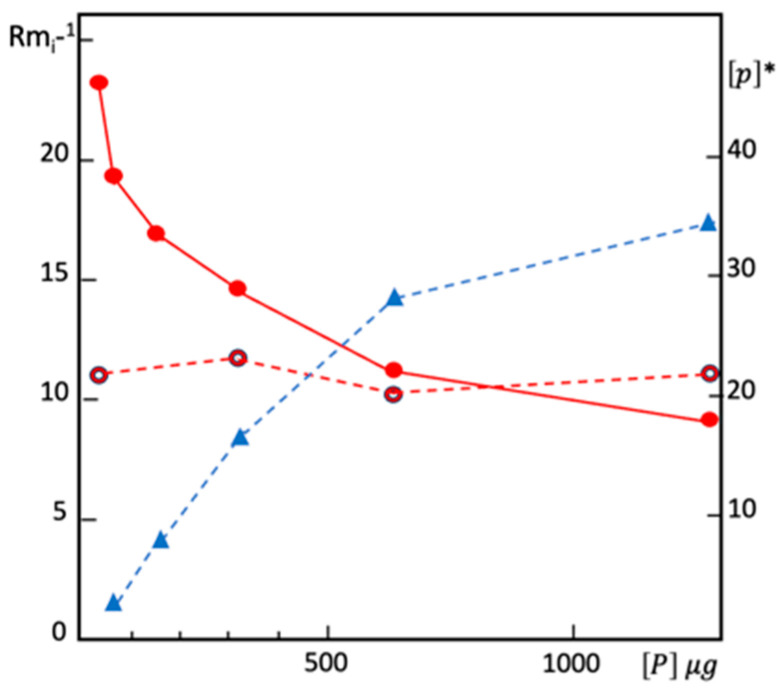
Reciprocal of relative mobility (*Rm*^−1^_i_) of a specific protein migrating in an affinity gel as a function of the amounts of proteins (μg) present in a complex biological medium. In the present case, the specific protein was human BChE tetramer G4 in the presence of different amounts of human plasma [*P*], and the immobilized ligand, [*L*] = 1.8·10^−4^ M, was m-amino-phenyl-trimethyl ammonium) (●); control gels: reciprocal of the relative mobility (*Rm*^−1^*_0_*) of the same enzyme samples in the absence of ligand [*L*] = 0 (◯); relative amounts of non-specific proteins interacting with immobilized-L blocked at the entrance of affinity gel (after Coomassie Brilliant Blue staining, the relative protein concentrations were determined by densitometric gel scanning and expressed as arbitrary intensity units [p]*) (▲). Adapted from an original figure in [42]; reproduced with permission of Elsevier.

Figure 13 illustrates the effect of competing proteins present in human plasma on the mobility of the BChE tetramer (G_4_) for a given concentration of immobilized ligand: as the concentration of proteins P in samples increases, the mobility of BChE also increases. In affinity gels, in the presence of the highest [*P*], *R_m,i_* becomes close to *R_m,0_*. In other words, because all ligand molecules are bound to competing proteins, the mobility of the tetramer is similar to its mobility in the absence of immobilized ligand (Figure 13). This statement is valid for all enzymes in all types of crude samples when the specificity of the immobilized ligand is promiscuous. In such situations, the total protein concentration [*P*] in complex samples must be as low as possible compared to [*L*] to minimize competing protein interferences that may impair specific interactions between the protein of interest and immobilized ligands (Figure 13). The activity detection method of the enzyme must be specific and as sensitive as possible.

#### 4.2.3. Detection of Protein/Enzyme Functionality

Alterations in protein conformation, such as pre-denaturation or partial unfolding, change the protein affinity for ligands and substrates and can be observed directly on electrophoretic gels after specific staining [1]. Similarly, changing the solvent, the physical conditions in AE, or incorporating stabilizers or denaturing agents into electrophoretic buffers may also result in changing binding affinities of proteins and enzyme activities in AE gels (cf. Figure 9). Single nucleotide variants of a protein may exhibit different binding affinities for a particular ligand, which can easily be detected from the variable protein mobilities (cf. Figure 11).

#### 4.2.4. Study of Protein Chemical Modifications

Chemical modifications affecting binding and active sites of proteins and enzymes may alter their binding affinities for specific ligands. Hořejší et al. [54] revealed that acetylation, sulfenylation, and photo-oxidation of lectins inhibit their hemagglutination activity but do not alter their affinity for the respective saccharides. Conversely, soman-phosphonylation of the S198 residue within the esteratic site (subsite of ChEs where acetylcholine is hydrolyzed to acetate and choline) of human BChE alters the enzyme binding affinity for immobilized procainamide (Figure 6) [44]. Although in this enzyme, the esteratic and ligand binding sites are distinct [66], small changes in conformational stability and flexibility of the active site gorge due to the chemical modification of S198 [59] are sufficient to change the affinity of the enzyme for procainamide.

Post-translational chemical modifications can also be detected and analyzed by AE. For example, the hydrophobic character of glycosyl-phosphatidyl inositol (GPI)-anchored dimeric forms of bee head acetylcholinesterase (AChE) was revealed by PAG-AE on immobilized acryloyl-6-amido-caproic acid N-octadecylamine [46]. Because GPI contains long fatty acid chains, AE on this hydrophobic affinity gel allowed us to quantify the hydrophobic nature of the anchor in terms of apparent *K_D_*. Differently glycosylated isoforms of proteins can be easily separated by AE on immobilized lectins [94].

#### 4.2.5. Study of Supramolecular Conjugates

**Figure 14:**
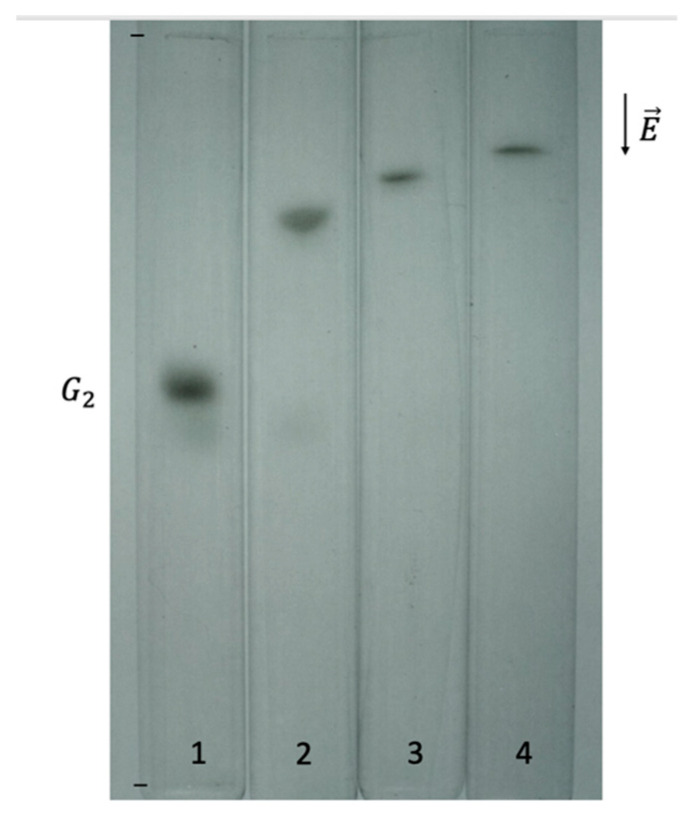
Affinity electrophoresis of the G_2_ form of human BChE in gels containing an immobilized octadecyl ligand (adapted from [96]). The concentration of immobilized ligand (μM) in gels 1 to 4 was: 1: 0; 2: 1.81; 3: 3.63; and 4: 7.25. Gels were stained for BChE activity. K_D_ for immobilized octadecyl ligand was 0.8 μM for G_2_ and 1.1 μM for pure HSA.

Conjugation of a protein to another macromolecule may alter the affinity of this protein for a specific ligand, which may be detected by determining the affinity of the conjugated macromolecule for a specific ligand. For example, human BChE can form heterologous complexes and conjugates with other proteins [95]. The plasma BChE comprises four molecular globular forms, i.e., G_1_, G_2_, G_3_, and G_4_. Forms G_1_, G_3_, and G_4_ correspond to monomer, dimer, and tetramer, respectively, the latter being the major form. G_2_ represents a covalent dimer composed of G_1_ and human albumin (HSA) linked via a disulfide bridge. Only C_2_ binds the immobilized octadecyl chains with the same affinity as HSA (Figure 14) on AE gels containing immobilized octadecyl-containing PA chains [96]. Then, it was shown that only G_2_ binds specific HSA antibodies. In addition, AE of G_2_ on block-copolymerized Cibacron blue F3GA showed that G_2_ tightly binds this dye much like HSA [46].

The C_5_ isoenzyme of human BChE is found in Caucasians at a frequency of about 10%. It is a hybrid protein composed of a BChE tetramer G_4_ non-covalently associated with a 60 kDa subunit (X). EA on hydrophobic-ligand-containing gels revealed the marked hydrophobic character of this subunit X [97]. However, the subunit X was only identified 27 years later by combining genetic linkage analysis, immunoblotting, and mass spectrometry analysis of polypeptide fragments. X is a polyproline-rich protein, lamellipodin, encoded by the last exon of the RAPH1 gene [98].

## 5. Affinity Capillary Electrophoresis

ACE has long been considered a powerful and fast tool for investigating protein kinetics and equilibrium binding constants, binding stoichiometry, and, more recently, high-throughput screening of vast ligand libraries.

Several excellent reviews on the ACE basic principles and technological issues have been published [77,99,100]. Different modes of ACE, including mobility shift displacement, pre-equilibrium ACE, kinetic ACE, and partial filling ACE, have been utilized for the study of protein–ligand binding in terms of binding and rate constants, stoichiometry, energy change, and reversibility of the reactions, which were thoroughly covered in the reviews [23,101]. Herein, we will limit our remarks to theoretical principles at the basis of measuring P-L binding affinity and most attractive recent trends. The different theoretical approaches can be applied to relate the changes in electrophoretic mobility (*μ*) of an injected protein upon binding the ligand (*L*) in the migration buffer filling the capillary.

These approaches consider the impact of endo-electroosmotic flow on the electrophoretic mobility [102,103], viscosity, pH, and ionic strength of buffers [104], and the adsorption of ligands and proteins on the capillary wall. Coating of the inner wall prevents adsorption [105]. Regarding 1:1 binding stoichiometry, binding constants (*K_A_*) can be estimated from the effective mobility (*μ_eff_*) of the ligand [106]:

(22) $$\mu_{eff}=\frac{\mu_{f}+\mu_{c}K_{A}\left[P\right]}{1+K_{A}\left[P\right]}$$

where *μ_f_* is the mobility of the free ligand; *μ_c_* is the mobility of the PL complex; and [*P*] is the protein concentration. In ACE, the concentration of protein must be much higher than the ligand concentration. Unlike gel AE, studying the binding kinetics by ACE is easier, and determining first- and second-order kinetic constants *k_a_* and *k_d_* (c.f. Equation (23)) is more straightforward [100,107].

(23) $$\;k_{a}\;P+L⇌PL\;\;k_{d}$$

ACE exhibited great potential for high-throughput screening of protein–ligand libraries and could be of interest for screening pharmacologically interesting enzyme SBIs.

ACE can be coupled to mass spectrometry [108] and other highly sensitive biophysical tools such as microscale thermophoresis [109]. Working with capillaries housed in liquid-thermostated cartridges at different temperatures up to 95 °C enables assessing the thermodynamic parameters of ligand binding and functional stability of enzymes [110].

## 6. Conclusions

Although modern techniques like isothermal titration calorimetry [111] and surface plasmon resonance [112] are fast and accurate for quantifying protein binding affinity, they require sophisticated and expensive equipment. On the contrary, AE is an appealing technique due to its simple principle, feasible synthesis of macro-ligands, and ease of realization in PA gels, using relatively simple and ubiquitous electrophoresis apparatuses. Moreover, it is ultra-sensitive, particularly for enzymes that act on chromogenic or fluorogenic substrates. On the other hand, a few obstacles limit its use in quantitative studies, such as difficulties in producing homogenous gels and problematic determination of the effective concentration of immobilized ligands. Otherwise, it is a powerful tool for comparative and qualitative studies of protein binding affinity.

The development of ACE (CZAE) has temporarily eclipsed interest in AE; the former has considerably expanded the applicability field of electrophoretic techniques in protein binding studies. The renewal of gel AE through ultra-miniaturization, specifically on micro-chip devices [113], and coupling to highly sensitive biophysical tools, such as mass spectrometry, has again thrown gel AE on the front line.

## Abbreviations

The following abbreviations are used in this manuscript:

AC	Affinity chromatography
ACE	Affinity capillary electrophoresis
AE	Affinity electrophoresis
BChE	Butyrylcholinesterase
BSA	Bovine serum albumin
ChE	Cholinesterase
CZAE	Capillary zone affinity electrophoresis
CZE	Capillary zone electrophoresis
HSA	Human serum albumin
IEF	Isoelectric focusing
PAG	Polyacrylamide gel
PAGE	Polyacrylamide gel electrophoresis
PEG	Polyethylene glycol
pI	Isoelectric point
SBI	Slow-binding inhibitor
